# Supplementary material for: Is Running Power a Useful Metric? Quantifying Training Intensity and Aerobic Fitness Using Stryd Running Power Near the Maximal Lactate Steady State
Source: Sensors (Basel). 2023 Oct 26;23(21):8729. doi: 10.3390/s23218729 (PMC10649254; doi:10.3390/s23218729)
Supplement: Supplementary file 1 [file sensors-23-08729-s001.zip › sensors-2662249-supplementary.pdf]

Supplementary Table S1. Mean physiological and perceptual responses to exercise near the maximal lactate steady state (MLSS), and indices of reliability between two runs at the MLSS.

	5% below MLSS		*At MLSS*		*5% above MLSS*		**ANOVA (DxI, D, I)**	*At MLSS (repeat)*		*Reliability of repeated runs at MLSS (30-min)*			
	**10 min**	**30 min**	**10 min**	**30 min**	**10 min**	**30 min[1]**		**10 min**	**30 min**	**t test (p)**	**Bias**	**LOA**	**ICC**
**$\dot{V}CO_2$ (L·min^{-1})**	2.76 [0.57]*	2.72 [0.56]*	2.92 [0.55]	2.88 [0.53]	3.14 [0.58] *,†	3.15 [0.57] *,†	0.104, 0.170, <**0.001**	2.90 [0.51]	2.87 [0.48]	0.597	0.02	−0.24 to 0.28	0.98 (0.95 to 0.99)
**RER**	0.88 [0.05]^{a,b}	0.86 [0.05]^{c}	0.89 [0.03]^{b}	0.87 [0.03]^{c}	0.92 [0.05]^{a}	0.91 [0.05]^{a,d}	**0.036, <0.001, 0.003**	0.89 [0.04]	0.88 [0.04]	0.391	−0.01	−0.07 to 0.05	0.81 (0.44 to 0.94)
**$\dot{V}_E$ (L·min^{-1})**	91.6 [17.9]^{a}	96.5 [19.1]^{b}	99.8 [17.5]^{c}	107.6 [17.9]^{d}	107.3 [17.9]^{e}	122.6 [21.2]^{f}	**<0.001, <0.001, <0.001**	97.6 [17.2]	105.5 [17.4]	0.198	2.1	−9.9 to 14.1	0.97 (0.90 to 0.99)
**HR (bpm)**	161 [9]^{a}	168 [9]^{b}	165 [8]^{c}	174 [8]^{d}	169 [7]^{e}	178 [8]^{f}	**0.006, <0.001, <0.001**	164 [9]	173 [9]	0.084	2	−5 to 9	0.94 (0.82 to 0.98)
**[BLa] (mM)**	2.8 [1.3]^{a}	3.2 [1.4]^{b}	3.7 [1.3]^{c}	4.3 [1.4]^{d}	5.2 [1.5]^{e}	7.4 [1.7]^{f}	**<0.001, <0.001, <0.001**	3.6 [1.5]	4.3 [1.5]	0.868	0	−2.1 to 2.2	0.85 (0.55 to 0.95)
**RPE (6-20)**	11 [1]^{a}	14 [2]^{b}	12 [2]^{c}	15 [2]^{d}	13 [2]^{e}	18 [1]^{f}	**<0.001, <0.001, <0.001**	11 [2]	15 [2]§	0.028	1	−2 to 3	0.84 (0.45 to 0.95)

DxI, duration by intensity; D, duration; I, intensity; LOA, limits of agreement; ICC, intraclass correlation; $\dot{V}CO_2$, carbon dioxide production; RER, respiratory exchange ratio; $\dot{V}_E$, ventilation; HR, heart rate; [BLa], blood lactate concentration; RPE, rating of perceived exertion. [1]Or the final 2 min if task failure was < 30 min.

For variables with a significant interaction effect, means at two timepoints within an intensity and means at the same timepoint across intensities are significantly different if they do not share a common letter (means at different timepoints across intensities were not compared). For other variables, the * denotes a significant difference from MLSS ($p < 0.05$), the † denotes a significant difference from

5% below MLSS ($p < 0.05$), and § denotes a significant difference between 30-min MLSS and 30-min repeat trial at MLSS measures ($p < 0.05$). Data are reported as mean [standard deviation]. n=15 for all variables.
